# Understanding the Steps Toward Mobile Early Intervention for Mothers and Their Infants Exiting the Neonatal Intensive Care Unit: Descriptive Examination

**DOI:** 10.2196/18519

**Published:** 2020-09-22

**Authors:** Kathleen M Baggett, Betsy Davis, Susan H Landry, Edward G Feil, Anna Whaley, Alana Schnitz, Craig Leve

**Affiliations:** 1 Mark Chaffin Center for Healthy Development School of Public Health Georgia State Universtiy Atlanta, GA United States; 2 Oregon Research Institute Eugene, OR United States; 3 University of Texas Health Sciences Center- Houston Houston, TX United States; 4 University of Kansas Kansas City, KS United States; 5 Juniper Gardens Children's Project University of Kansas Kansas City, KS United States

**Keywords:** early intervention, equity, NICU, low birthweight, transition, mobile internet intervention, infants, mothers

## Abstract

**Background:**

Neonatal intensive care unit (NICU) history, combined with systemic inequities for mothers of nondominant cultures and mothers who are socioeconomically disadvantaged, places infants at an extraordinary risk for poor developmental outcomes throughout life. Although receipt of early intervention (EI) is the best single predictor of developmental outcomes among children with and at risk for early developmental delays, mothers and infants with the greatest needs are least likely to receive EI. Mobile internet-based interventions afford substantial advantages for overcoming logistical challenges that often prevent mothers who are economically disadvantaged from accessing EI. However, the bridge from the NICU to a mobile internet intervention has been virtually unexplored.

**Objective:**

This study aims to examine progression flow from NICU exit referral to an early mobile internet intervention to increase EI access and promote parent mediation of infant social-emotional and communication development.

**Methods:**

Three NICUs serving the urban poor in a Midwestern city were provided support in establishing an electronic NICU exit referral mechanism into a randomized controlled trial of a mobile internet intervention for mothers and their infants. Measurement domains to reflect the bridge to service included each crucial gateway required for navigating the path into Part C EI, including referral, screening, assessment, and intervention access. An iterative process was used and documented to facilitate each NICU in establishing an individualized accountability plan for sharing referral materials with mothers before their NICU exit. Subsequent to the referral, progression flow was documented on the basis of a real-time electronic recording of service receipt and contact records. Mother and infant risk characteristics were also assessed. Descriptive analyses were conducted to summarize and characterize each measurement domain.

**Results:**

NICU referral rates for EI were 3 to 4 times higher for open-shared versus closed-single gatekeeper referral processes. Of 86 referred dyads, 67 (78%) were screened, and of those screened, 51 (76%) were eligible for assessment. Of the 51 assessment-eligible mothers and infants, 35 dyads (69%) completed the assessment and 31 (89%) went on to complete at least one remote coaching intervention session. The dyads who accessed and engaged in intervention were racially and ethnically diverse and experiencing substantial adversity.

**Conclusions:**

The transition from the NICU to home was fraught with missed opportunities for an EI referral. Beyond the referral, the most prominent reason for not participating in screening was that mothers could not be located after exiting the NICU. Stronger NICU referral mechanisms for EI are needed. It may be essential to initiate mobile interventions before exiting the NICU for maintaining post-NICU contact with some mothers. In contrast to a closed, single point of referral gatekeeper systems in NICUs, open, shared referral gating systems may be less stymied by individual service provider biases and disruptions.

## Introduction

### Background

Low infant birth weight, requiring neonatal intensive care unit (NICU) treatment, places infants at high risk for a host of detrimental outcomes, including cognitive, language, and social delays and disabilities, which often persist into adulthood [1-3]. The discharge from a NICU is a stressful transition in which the responsibilities for around-the-clock care of a fragile infant shift from a NICU medical team to parents. This transition is particularly burdensome for mothers who often experience the bulk of responsibility for infant care [4]. A central challenge of this transition is to connect families with early intervention (EI) services.

Parent participation in EI services is the single best predictor of developmental outcomes for children with and at risk for developmental disabilities identified during the first year of life [5,6]. EI can support parents in recognizing and responding sensitively to subtle cues of infants with developmentally immature social signaling systems, which is foundational for establishing social interaction, feeding, and sleeping routines that promote infant regulation and social communication competency development [7]. By and large, however, the reach of EI services that target parent practices to support early social-emotional and communication competencies is extremely limited relative to societal needs [6,8,9]. Moreover, substantial inequities persist in EI access. Infants whose mothers are socioeconomically disadvantaged and of nondominant cultures are disproportionately over-represented in NICUs in the United States [10] because of historically driven systemic and structural inequities [11]. However, Part C service systems disproportionately serve White, middle-income, and upper-middle-income families [12].

In contrast to White infants in the United States, African American infants with special needs are five to eight times less likely to be referred for EI services [12]. They are also more likely to receive lower-quality care in NICUs because of both structural and interpersonal racism [11]. Structural barriers such as low-paying, unstable work with unpredictable hours without paid leave or quality childcare can deplete mothers’ physical, psychological, and social resources for parenting a newborn in general and for engaging in the NICU in particular [13,14]. Implicit bias faced by these mothers within the medical system [15] can exacerbate maternal stress, shown to continue long after the NICU experience [16], and undermine mothers as central change agents of their infants’ development and well-being. As social determinants of health, these inequities threaten infant development by compromising maternal functioning and parenting practices. Moreover, it is possible that these inequities lessen the willingness of mothers to connect with future intervention services [17], delay intervention access, and decrease mothers’ opportunities to access and benefit from EI to improve infant developmental outcomes.

Recognizing the substantial impact of systematic gaps in access to timely intervention, Child Find efforts emphasize the need to identify, locate, and assess all infants with developmental delays, particularly those who are poor and of nondominant culture [6]. Unfortunately, published research on Child Find efforts, which are crucial for receiving EI services, tends to reflect a striking absence of representative samples of these families [6]. Published studies that systematically examine crucial junctures at which mothers either progress toward or fall off the pathway from NICU referral to EI access are also lacking. To obtain EI subsequent to NICU exit, mothers must successfully navigate crucial gateways that bridge the NICU experience to EI service receipt. These gateways include referral, screening, assessment, and intervention access [18], each of which must be navigated successfully to obtain EI. Early systemic barriers to intervention include failure to provide supported referrals, lack of routine developmental monitoring and screening, and insufficient reach of public awareness campaigns about the relevance of EI for infants and toddlers and their families [6,18]. The cost of home visiting intervention programs is particularly prohibitive because of state budget crises that often result in the rationing of state-funded home visiting services [9,19]. When home visiting programs are available, the barriers to parent engagement include unpredictable work schedules; shift work outside the 9 to 5 workday; transient housing; and living with relatives, friends, or landlords who are gatekeepers to the home and unamenable to home visits [20].

Mobile internet interventions, particularly those with remote coaching, afford substantial advantages for overcoming logistical challenges that often prevent mothers who are poor and of nondominant culture from accessing EI [20]. The advantages of mobile internet interventions include around-the-clock accessibility to program content, greater ease and flexibility in scheduling and rescheduling remote visits, less stigma, and greater parental autonomy to select and share video-recorded interactions at any time of day for the purpose of obtaining EI support [20]. Although advantages for mobile interventions are known to exist, the bridge from a NICU exit referral to a mobile internet intervention has been virtually unexamined in published studies.

### Objectives

To increase equitable EI access, the purpose of this paper is to examine progression flow from a NICU exit referral to an early mobile internet intervention to promote parent mediation of infant social-emotional and communication competency development.

The progression flow on the bridge from the NICU to EI access is viewed within a randomized controlled pilot study of a mobile internet intervention with remote coaching designed to strengthen parent practices, which scaffold infant social-emotional and communication competencies. To illuminate the junctures at which mothers connect with or disconnect from progressing from the NICU to EI, we address the following questions: (1) What NICU referral structures impede or facilitate referral to intervention? For example, the diffusion literature suggests that when responsibility for action is shared across multiple members of a group, it can result in reduced outcome monitoring, a reduced sense of individual agency, and diffusion of responsibility and action [21]. (2) Among mothers referred for intervention, what is the screening rate, and what are the identified reasons for failure to screen? (3) Among mothers screened, what is the assessment completion rate, and what are the identified reasons for failure to assess? (4) Among mothers and infants assessed, what is the rate of intervention initiation and completion of the core intervention? and (5) What are the demographic and risk characteristics of mothers who traverse the bridge from NICU referral to intervention access? We hypothesize that when mothers are supported through each gateway on the bridge from the NICU to EI, the resultant internet-based intervention sample will be diverse with regard to demographic and risk characteristics.

## Methods

### Procedures

Our mobile internet intervention study procedures, from a NICU exit referral to a mobile intervention, provide a unique framework for examining progression flow through the crucial junctures that mirror the Part C EI system gateways: referral, screening, assessment, and intervention [18]. After institutional review board approval, recruitment efforts focused on 3 Level 3 NICUs serving the urban poor in a Midwestern city. These NICUs were selected because they were part of a medical conglomerate with similar characteristics that included a centralized geographic location in the urban core within 2 square miles of one another, similar annual admission rates, and a racially and ethnically diverse patient population, including those who lack insurance and the ability to pay. Through an iterative process, the research team conducted a series of meetings with each NICU team to generate a referral accountability plan, which was documented by the research team and provided to the NICU team for review and revision until the NICU team confirmed that their plan was complete and accurate. Each NICU-generated plan specified the NICU personnel who would share referral information with mothers, collect cards that mothers signed indicating their interest in being contacted by the study team, send electronic referrals to the research team, and respond to a biweekly prompt to provide an electronic referral update. Electronic referral update reports included the number of eligible mother-infant dyads in the NICU during the most recently completed referral period, the number of mothers with whom referral information was discussed, the outcome of each referral discussion, barriers to referral, and identified solutions. The research team provided referral materials to each NICU, which included service provider posters with mother and infant eligibility criteria to remind and prompt providers to refer all eligible mothers, a mobile intervention study letter to be shared by providers with mothers, a mother interest card for mothers to grant permission for study team follow-up, and a script for providers to use when sharing referral materials and collecting mother interest cards. An electronic NICU referral mechanism was established for NICU service providers to connect mothers and their low-birth-weight infants to a randomized controlled trial of a mobile internet intervention.

Referral criteria included biological or adoptive mothers, living in the metropolitan area of the NICU, who spoke English and whose infants at birth weighed <2500 g, were at least 24 weeks’ gestational age, were no more than 5 months corrected gestational age at NICU exit, and who were not diagnosed with hydrocephalus, bronchopulmonary dysplasia, or beyond a grade 3 intraventricular hemorrhage. Referral criteria were established to avoid potential study burden for mothers of infants who were experiencing acute medical crises, including a high risk for NICU return or intensive care unit (ICU) entry. NICU teams were encouraged to refer all eligible mothers and infants in addition to any and all other service referrals such that all referred mothers were free to participate in existing community service referrals as usual without exclusion.

On receipt of each electronic referral, the research staff recorded the date of referral, referral source, and referral contact information into a project database. Research staff mailed consent forms to referred mothers and contacted them by phone to (1) confirm referral eligibility criteria, (2) review and discuss the consent form, and (3) determine whether mothers viewed themselves as able and willing to engage in the intervention study. Mothers who could not be reached by phone because of a disconnected number or failure to connect after at least five attempts were sent a letter encouraging mothers to contact the study team if interested in the program. Mothers’ perceived ability to participate in the study was determined on the basis of their negative responses to a brief structured interview question in which mothers were first informed of personal situations that should be prioritized over intervention study participation, such as homelessness, shelter residence, inpatient mental health or substance abuse treatment, or a major physical or mental illness requiring intensive treatment such as schizophrenia, cancer, or HIV/AIDS. Mothers were then asked whether they were experiencing one or more of these situations or any other situation that could interfere with their ability to participate in the intervention study. An affirmative response was exclusionary and met the criteria for intervention study ineligibility. For mothers who were screened eligible and agreed to participate in the study, an in-home assessment visit was scheduled. All contact attempts, the outcome of each contact attempt, the eligibility screening outcome, and the scheduled assessment date were recorded in the project database.

#### Assessment

Informed consent was obtained at the onset of a 2-hour, in-home assessment visit. Electronic questionnaires were completed by mothers on the web via Qualtrics entry on an iPad (Apple Inc) to provide information about demographics and maternal and infant risk characteristics. The *Measurement Domains and Measures* Section provides a full description of the measurement domains and measures. Assessments were conducted by research assistants who had obtained at least a bachelor’s degree in education, human development, or psychology and had at least 2 years of intervention research experience conducting in-home assessments and mobile intervention protocols with mothers and infants. Assessors were trained and observed to implement the assessment protocol with fidelity before data collection. The assessment details are also provided in the *Measurement Domains and Measures* section.

#### Mobile Intervention

Following assessment, mothers were randomized to 1 of 2 mobile internet interventions with identical structures. For both groups, the number of sessions and structural components of each session included (1) a web-based self-directed learning program through video-based teaching with check-in questions and provision of immediate corrective feedback, (2) an action plan outlining daily activity practice (homework) based on session content, (3) parent-recorded video and secure upload of session skill practice during interaction with her infant, and (4) a video-based coach call to coview the parent-recorded video of interaction with her infant [20]. For both intervention groups, meaningful access to a mobile internet intervention was operationalized as (1) mothers’ completion of an in-home intervention session in which mothers were fully guided and scaffolded to interact with each mobile intervention component (ie, video modeling content, review questions, action plan, video creation, and coach call) and (2) mothers’ completion of each of the above content components of the remote intervention session with on-demand scaffolding provided through messaging, phone, or video call.

At the in-home intervention orientation visit, all mothers were given an iPhone with unlimited data, text, and call plan. They were granted entry into a 12-session mobile internet intervention. Coaches used a demonstration video to introduce mothers to the mobile intervention, use the mobile phone features, and navigate through the first mobile intervention session. Coaches verbally scaffolded mothers’ use of each session component by providing the phone and materials to the mother and serving as a guide on the side when mothers navigated through the entire first session, including the coach call procedures.

After the first session with coach guidance and full scaffolding, mothers autonomously completed the second intervention session with on-demand remote coach scaffolding between and during coach calls. The demand context for the coach response included (1) questions from the mother and (2) coach electronic monitoring of mothers’ progress or nonprogress through intervention session components and feedback to celebrate mothers’ successes and address barriers to progress. We expected that this meaningful access support in the first 2 sessions would increase the probability of mothers’ continued progress in completing the remaining 10 remote sessions.

### Measurement Domains and Measures

The measurement domains included the following: (1) challenges and solutions to referral of mothers and infants from the NICU, (2) mother and infant progression flow from the point of referral through the point of intervention access, and (3) maternal and infant demographics and risk characteristics. Measures pertaining to each domain are identified below.

#### Challenges and Solutions

Challenges and solutions to referral of mothers and infants from the NICU were documented by research assistants based on a review of biweekly NICU referral reports, follow-up discussion of barriers and solutions with NICU teams, and recorded events observed by the research team, such as changes in the NICU personnel, NICU referral strategy changes, and reported beliefs of the NICU personnel about referral.

#### Progression Flow Data

Progression flow data included records of electronic referrals and documentation of attempted screening calls, completed screening calls, screening outcomes, assessments scheduled, electronic recorded time stamp of assessment completion, intervention session completion, and recorded time of coach call completion.

#### Demographics

Demographic information included data on mother and infant age (years and months, respectively), ethnicity, race (based on federal reporting categories), mothers’ educational level (multiple categories ranging from less than high school to postgraduate), no significant other relationship status, annual household income (6 categorical ranges), and number of children and adults in the household.

#### Maternal and Infant Characteristics

Maternal and infant risk characteristics included maternal financial strain and depression, infant time in the NICU, birth weight in ounces for calculation of very low birth weight status, months premature, corrected gestational age, infant social-emotional development concerns, and social-emotional behavior challenges (see Measures section below). Financial strain was measured using a 9-item questionnaire with a 5-point Likert-type scale for difficulty paying bills, money left over after paying bills, and money availability for necessities and other activities [22]. Maternal risk for postpartum depression was assessed using the Postpartum Depression Screening Scale (PDSS) [23]. The PDSS is a 35-item Likert-type self-report instrument that demonstrates strong sensitivity and specificity for postpartum depression in the 15 months after childbirth [24]. It has adequate psychometrics for mothers of infants in NICUs [25].

Infant social-emotional development was assessed using Ages & Stages Questionnaires: Social Emotional (ASQ-SE) screening tool [26]. This brief screener of social emotional functioning demonstrates high internal consistency with an overall α of .82 [27].

Infant social-emotional behavior concerns were assessed with the Devereux Early Childhood Assessment for Infants (DECA-I) scale [28]. The DECA-I is a 33-item behavior rating scale that assesses child protective factors central to the social and emotional health and resilience of infants from 1 to 18 months. This norm-referenced measure, with demonstrated adequate reliability and validity [29], provides a cutoff score for social-emotional behaviors in the concern range.

## Results

To address the first research question, “What NICU referral approaches impede or facilitate referral from the NICU to a mobile EI?” we provide a brief description of the NICU site referral processes and referral rates as well as a summary of identified factors that impeded or facilitated referral. The established referral processes for each NICU site, as noted above, were identical with regard to the content of printed referral material for NICU personnel and mothers, inclusion and exclusion criteria for referral, electronic referral mechanism, and biweekly NICU referral prompting and reporting. However, there were systematic differences in the ways that each NICU self-selected to engage NICU personnel in their referral approach and to adhere to their established referral plan. Sites 1 and 2 established a single gatekeeper as a point of referral from the NICU to the intervention. In both cases, the gatekeeper was the social worker responsible for patient discharge. This individual also responded to biweekly prompts for site referral accountability reporting. Hence, we refer to Sites 1 and 2 as a closed, single gatekeeper approach. In contrast, Site 3 established an open, shared referral approach in which the NICU psychologist shared referral materials with all nursing and social work staff and encouraged conversations between staff and mothers about the referral materials, including whether mothers had seen the materials, what questions they had about the referral, if they had already expressed interest in referral to learn about the intervention, or would like to be referred to learn about the opportunity. The NICU psychologist also engaged in conversations with mothers about study referral and served as the contact for responding to biweekly accountability prompts and reporting.

**Figure 1:**
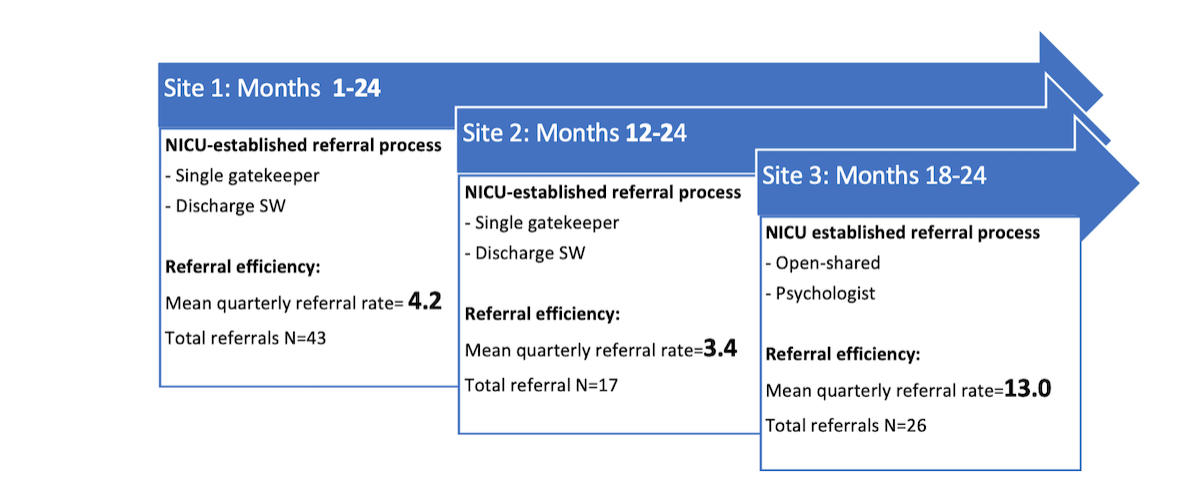
Quarterly referral rates by NICU referral site. NICU: neonatal intensive care unit; SW: software.

The 3 NICU sites referred to a combined total of 86 mothers and their infants for mobile intervention, with Site 1 contributing 43 referrals (50% of the referral sample) within a 24-month period, Site 2 contributing 17 referrals (20% of the referral sample) within a 12-month period, and Site 3 contributing 26 referrals (30% of the referral sample) within a 6-month period. Sites were added sequentially such that the referral window length varied with a 24-month referral window for Site 1, a 12-month window for Site 2, and a 6-month referral window for Site 3. As noted above, the annual census of Level 3 NICUs was similar. Examination of mean quarterly referral rates in Figure 1 shows that Sites 1 and 2 had a much lower mean quarterly rate of 4.2 and 3.4 referrals per quarter, respectively, in contrast to Site 3 with 13 referrals per quarter.

A review of the referral documentation yielded several factors associated with lower versus higher quarterly referral rates. These included the NICU self-selected referral approach and adherence to site-generated referral plans. In contrast to the open, shared referral structure, the closed single gatekeeper referral sites demonstrated the lowest quarterly referral rates and less adherence to their site-generated referral plan. Examples of challenges to adherence included the inability to refer as planned because of personnel issues such as illness, unpredictable staffing patterns, and staff turnover. Another challenge to adherence was provider belief that referral is best governed by provider clinical judgment rather than the principle of universal referral of all eligible infants and mothers. In contrast, the open, shared referral site demonstrated stronger adherence to the referral plan, which yielded the highest referral efficiency rates of the 3 sites.

**Figure 2:**
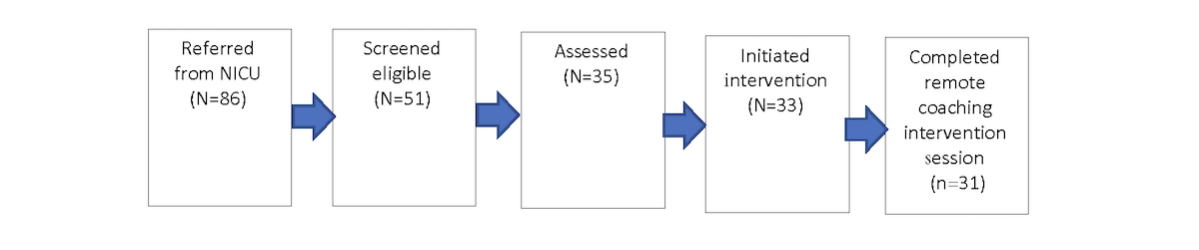
Mean quarterly referral rate.

Herein, we address research questions 2 to 4 pertaining to the junctures at which mothers and their infants either fall away from or progress through the crucial sequential gateways of screening, assessment, and intervention access on the path from referral from the NICU to early mobile intervention engagement. As displayed in Figure 2, 86 mothers referred from the NICU for mobile intervention, 67 (78%) were screened. Seven mothers (8%) declined to be screened and 12 mothers (14%) could not be reached for complete phone screening. The most common reasons for failure to contact included incomplete or inaccurate referral contact information, phone disconnection, and returned mail. Of the 67 mothers screened, 51 (76%) were eligible for assessment. Of the 51 screened eligible, 10 mothers (20%) could either not be contacted to schedule an assessment or could not be assessed because of a family move or infant complications requiring ICU or other hospitalizations, 6 (12%) declined assessment, and 35 mothers (69%) and their infants initiated assessment.

Of the 35 mothers who initiated the assessment, 1 mother died and another moved before initiating intervention. The remaining 33 mothers initiated intervention, with 31 mothers completing at least one remote coaching session. After initiating the intervention, reasons for failure to complete at least 1 remote session included a maternal death and 1 mother moving to an undisclosed location to escape neighborhood and partner violence. Of the 35 mothers assessed, 31 (89%) meaningfully accessed intervention by completing at least one intervention session with a remote coaching call. Examination of intervention access and engagement patterns showed that there were no significant differences between the intervention groups. On average, mothers completed 9.18 intervention sessions (all core content). The modal number of sessions completed was 12, which constituted all possible sessions.

Finally, we describe the demographic and risk characteristics of mothers and their infants who accessed the intervention. It was anticipated that when mothers were supported at each gateway, the resultant intervention sample would be ethnically and racially diverse. In addition, we expected the sample to reflect a high level of need relative to socioeconomic stressors and distress. We first examined demographic and risk characteristics by intervention group, which resulted in the finding of no significant between-group differences in any of the mother-infant demographic or risk characteristics. Hence, sample characteristics are presented for the combined intervention groups in Table 1. The sample was racially and ethnically diverse, with 55% (18/33) infants identified by their mothers as Black and 21% (7/33) identified as Latinx. The sample that accessed and engaged in the intervention was highly vulnerable. The majority of mothers were experiencing significant psychosocial stressors.

For the majority (81%), income was at <300% of the Federal Poverty Guideline, with 50% of the sample at or below 100% of the Federal Poverty Guideline. Relative to financial strain, 92% of mothers reported “not enough or barely enough” money left at the end of the month after paying bills. The majority did not have a college degree, and nearly half (43%) had not graduated from high school. More than half of the mothers were experiencing significant symptoms of depression and reported no significant other relationship. More than one-third of infants obtained scores classified in the concern range for social-emotional development and behavior on the ASQ-SE and the DECA-I Toddler, respectively.

**Table 1:** Sample demographics and risk characteristics of the mobile intervention access sample.

Variable				Value
**Maternal**				
	Age (years), mean (SD); range		27.03 (5.49); 17.67 to 38.00	
	**Race/ethnicity, %**			
		Black		52
		Latinx		21
	Maternal education (<college degree), %		88	
	**Income, %**			
		≤100% federal poverty level		50
		101%-300% federal poverty level		31
		>300% federal poverty level		19
	Relationship status (no significant other)		52	
	Significant depressive symptoms^a^ (>PDSS^b^ clinical cutoff)		58	
**Infant**				
	Gender (female), %		49	
	**Race/ethnicity, %**			
		Black		55
		Latinx		21
	**Birth weight**			
		Mean (SD); range, g		1859.04 (988.20); 510.29 to 4053.98
		Extremely low, %		18
		Very low, %		24
		Low, %		39
		Non-LBW^c^ complication, %		18
	Prematurity level (months), mean (SD); range		1.60 (1.13); −0.72 to 3.48	
	Time in NICU^d^ (months), mean (SD); range		1.36 (1.15); 0.16 to 4.70	
	Chronological age at pre (months), mean (SD); range		4.01 (2.33); 0.45 to 10.32	
	Gestational age at pre (months), mean (SD); range		2.43 (2.26); −0.72 to 8.74	
	Social-emotional functioning^a^		39% ASQ-SE^e^ developmental concern; 36% DECA^f^ behavioral concern	

^a^To establish significant symptoms of maternal depression, infant social-emotional developmental functioning concern, and behavioral concern, established clinical cutoff scores were used for the PDSS total depression score, ASQ-SE, and DECA, respectively.

^b^PDSS: Postpartum Depression Screening Scale.

^c^LBW: low birth weight.

^d^NICU: neonatal intensive care unit.

^e^ASQ-SE: Ages & Stages Questionnaires: Social Emotional.

^f^DECA: Devereux Early Childhood Assessment.

## Discussion

The 3 referring Level 3 NICUs of similar annual census size and located within 2 square miles of one another demonstrated different levels of referral efficiency. In contrast to the closed, single gatekeeper referral approach of 2 NICUs, the shared, open referral approach of the third NICU resulted in higher referral efficiency. In addition, the shared, open NICU referral approach was associated with fewer reported disruptions in implementing their plan to discuss mobile intervention referral information with all eligible mothers. Within the closed single gatekeeper referral approach, the transition from the NICU to home was fraught with missed opportunities for EI referral. Not only were single gatekeeper NICUs more likely to report that their plans for referral were more often disrupted because of external factors such as unanticipated changes in staffing plans, but they also more often reported that they did not discuss referral information with mothers who they did not think would be interested in referral. This suggests that having a closed single gatekeeper referral system may be more susceptible to the bias of a single person’s judgment, which leads to missed opportunities for referral. In contrast, an open shared process involving multiple potential points of referral may afford more protection against individual bias that disrupts referral to EI. It is of interest to note that our approach to identify a NICU point of contact to interact with the study team and maintain oversight for the transfer of all internal NICU referrals was informed by the dissemination and implementation literature. This indicates the crucial role of identifying and engaging *champions* to support the establishment of implementation procedures [30]. Although each of our NICU points of contact self-identified as a *champion* of NICU referral into EI, only 1 of the 3 NICU points of contact provided an operational demonstration of *championing* as evidenced by actionable activities such as (1) communicating a shared responsibility of all NICU team members to engage in conversations with mothers about the importance of EI and (2) encouraging repeated and redundant opportunities for mothers to consider their own readiness to act on a referral for EI. It is likely that this type of operationalization of champions may play a crucial role in protection against the diffusion of responsibility within open, shared referral gating systems.

After referral, the most common reason for mothers and their infants to fall off the path toward intervention was that they could not be contacted after leaving the NICU. Hence, for some mothers, it may be important to conduct screening and assessment in the NICU to establish mobile intervention contact before the transition home. Several factors likely contributed to the inability to contact mothers after their transition home. In addition to the most commonly documented reasons, which included incomplete referral contact information and family mobility, another factor likely to have interfered with contact was an exacerbation of maternal distress that may have interfered with their ability to respond to contact attempts. The transition from the NICU to home is well documented as a time of heightened distress above and beyond the notable stress of NICU experience for many mothers [4]. To promote engagement in EI, it may be important for some mothers to establish supportive intervention contact, which can buffer against transition stress before the transition from the NICU to home.

Most screened eligible mothers and their infants (69%) selected to participate in and engaged in assessment, and 89% of those assessed went on to meaningfully engage in the mobile intervention. The fact that mothers completed, on average, at least 9 sessions, constituting all the core content, and that the modal pattern was the completion of all 12 content sessions is noteworthy. In contrast, home visiting studies of parenting interventions have consistently documented the concerning finding that, on average, approximately half of intended mothers receive any intervention and that the average amount of intervention received is, on average, only 25% of what was intended [29]. Intervention initiation and engagement in our mobile intervention sample were substantially higher. Moreover, this mobile intervention sample was racially and ethnically diverse and experienced significant psychosocial stressors. Hence, it is possible to engage mothers of nondominant culture and their infants who are experiencing a host of psychosocial stressors in a mobile EI program. However, there’s a need to establish stronger NICU referral mechanisms to EI.

### Limitations

The limitations of this research include a small convenience sample restricted to descriptive methods. In this small convenience sample, documented barriers to referral pertained to NICU referral characteristics. We did not have access to the NICU-level data about potential infant factors that could have influenced NICU health provider referral and/or parental acceptance of referral to EI. Our examination of maternal responses, in terms of moving toward or falling away from the path to intervention access, was conducted within an intervention study wherein resources, including ongoing training and support, were consistently applied to reinforce intervention research staff to prioritize and sustain outreach efforts in the face of substantial maternal and infant adverse experiences. Part C EI programs, especially with regard to resources for ongoing staff training and support, are often strained. Hence, the transferability of effective outreach strategies to facilitate maternal movement from referral to intervention access must take into account resource differences and work toward efforts to increase training and support resources within Part C EI programs if we are to succeed in reaching mothers and infants most in need.

### Implications for Future Research

Future studies should include NICU patient population-level data to examine infant characteristics that may be associated with referral provision and referral acceptance. To elucidate solutions for overcoming referral barriers within the NICU, future research needs to be conducted within NICUs to determine what factors in an open-gating system are associated with higher rates of referral such that these can be experimentally implemented and studied to increase effective and efficient referral practices. Subsequent research needs to be conducted with larger samples of NICUs to explicate characteristics of intervention referral champions and their operational execution of engaging NICU teams in processes that promote universal referral, characterized by broad, repeated, and redundant contact opportunities for referral. Dissemination and implementation of best practices identified from such research are crucial for improving equitable referrals such that all parents with infants in the NICU are provided opportunities to enter the first gateway on the path to accessing needed intervention, regardless of race, ethnicity, and income. Beyond NICU referral optimization, the resource infrastructure within Part C EI programs warrants closer examination with regard to the mechanisms that optimize or jeopardize family engagement at every crucial juncture on the pathway from referral to intervention access.

## Abbreviations

ASQ-SE: Ages & Stages Questionnaires: Social Emotional
DECA-I: Devereux Early Childhood Assessment for Infants
EI: early intervention
HHS: Health and Human Services
HRSA: Health Resources and Services Administration
NICU: neonatal intensive care unit
PDSS: Postpartum Depression Screening Scale
